# Predictors of outcome of noninvasive ventilation in severe COPD exacerbation

**DOI:** 10.1186/s12890-019-0892-9

**Published:** 2019-07-18

**Authors:** Alexandru T. Steriade, Shirin Johari, Nicoleta Sargarovschi, Daniela Necula, Cornelia E. Tudose, Diana Ionita, Miron A. Bogdan, Dragos Bumbacea

**Affiliations:** 1 Department of Pneumology & Acute Respiratory Care, “Elias” Emergency University Hospital, 17 Marasti Blvd, 011461 Bucharest, Romania; 2 “Carol Davila” University of Medicine and Pharmacy, 8 Eroii Sanitari Blvd, 050474 Bucharest, Romania; 3 “Marius Nasta” Institute of Pneumology, 90 Viilor St., București, 050152 Bucharest, Romania

**Keywords:** AECOPD, NIV duration, IPAP, Mortality, Left heart dysfunction

## Abstract

**Background:**

Noninvasive ventilation (NIV) reduces the rate of endotracheal intubation (ETI) and overall mortality in severe acute exacerbation of COPD (AECOPD) with acute respiratory failure and is increasingly applied in respiratory intermediate care units. However, inadequate patient selection and incorrect management of NIV increase mortality. We aimed to identify factors that predict the outcome of NIV in AECOPD. Also, we looked for factors that influence ventilator settings and duration.

**Methods:**

A prospective cohort study was undertaken in a respiratory intermediate care unit in an academic medical center between 2016 and 2017.

Age, BMI, lung function, arterial pH and pCO2 at admission (t0), at 1–2 h (t1) and 4–6 h (t2) after admission, creatinine clearance, echocardiographic data (that defined left heart dysfunction), mean inspiratory pressure during the first 72 h (mIPAP-72 h) and hours of NIV during the first 72 h (dNIV-72 h) were recorded.

Main outcome was NIV failure (i.e., ETI or in-hospital death). Secondary outcomes were in-hospital mortality, length of stay (LOS), duration of NIV (days), mIPAP-72 h, and dNIV-72 h.

**Results:**

We included 89 patients (45 male, mean age 67.6 years) with AECOPD that required NIV. NIV failure was 12.4%, and in-hospital mortality was 11.2%. NIV failure was correlated with days of NIV, LOS, in-hospital mortality (*p* < 0.01), and kidney dysfunction (*p* < 0.05). In-hospital mortality was strongly associated with days of NIV (OR 1.27, 95%CI: 1.07–1.5, *p* < 0.01) and with FEV1 (*p* < 0.05). All other investigated parameters (including left heart dysfunction, dNIV-72 h, mIPAP-72 h, pH, etc.) did not influence NIV failure or mortality. dNIV-72 h and days of NIV were independent predictors of LOS (*p* < 0.01). Regarding the secondary outcomes, left heart dysfunction and pH at 1-2 h independently predicted NIV duration (dNIV-72 h, *p* < 0.01), while BMI and baseline pCO2 predicted NIV settings (mIPAP-72 h, *p* < 0.01).

**Conclusion:**

In-hospital mortality and NIV failure were not influenced by BMI, left heart dysfunction, age, nor by arterial blood gas values in the first 6 h of NIV. Patients with severe acidosis and left heart dysfunction required prolonged use of NIV. BMI and pCO2 levels influence the NIV settings in AECOPD regardless of lung function.

## Background

Chronic obstructive pulmonary disease (COPD) is the third leading cause of death in the world. It has been responsible for 3 million deaths in 2016, and results in significant morbidity, with 63.5 million disability-adjusted life years (DALY) lost worldwide in 2016 [1]. Acute exacerbation of COPD (AECOPD) is a frequent complication of COPD that may result in hospitalization with major healthcare system and social costs and is responsible for increased morbidity and mortality [2].

Standard treatment of severe COPD exacerbation includes rapid-acting bronchodilators, systemic steroid, antibiotic, and controlled oxygen therapy [3]. Addition of non-invasive ventilation (NIV) to this regimen in patients with acute hypercapnic respiratory failure has resulted in reduced rates of endotracheal intubation (ETI) and overall mortality [4]. The development of respiratory intermediate care units has expanded the use of NIV in COPD exacerbation outside ICU [5]. However, incorrect patient selection at admission or under-recognition of NIV failure leads to delay of ETI which subsequently is associated with increased mortality, with reported failure rates that vary between 9 and 50% [6].

Potential causes of NIV failure might be lack of trained staff, over-reliance on NIV effectiveness, comorbidities, and lack of specific recommendations regarding optimal duration and settings of NIV [7].

NIV settings and duration are determined by the physician in charge based on expert opinion recommendations [7] and personal or group experience. There is a lack of published reports on determinants of NIV settings and duration and the influence of these parameters on outcomes like NIV failure or mortality.

Consequently, we aimed to evaluate known risk factors and to identify new such predictive indicators that influence NIV outcomes in our study population in a newly established RICU.

Main outcome was NIV failure (defined as endotracheal intubation or in-hospital death). Secondary outcomes were length of hospital stay (LOS), in-hospital mortality, NIV duration in hours during the first 72 h (dNIV-72 h), days of NIV and mean inspiratory pressure in the first 72 h (mIPAP-72 h).

## Methods

An observational prospective cohort study was undertaken in a respiratory intermediate care unit in an academic medical center for 16 months between 2016 and 2017. We included consecutive patients with severe COPD exacerbation and respiratory acidosis that were non-invasively ventilated with BPAP with volume assured pressure support (VAPS) mode.

### Non-invasive ventilation

Initiation and management of NIV were done according to an internal protocol compliant with international guidelines. In brief, non-invasive ventilation was instituted in patients which despite one hour of intensive medical treatment comprising controlled oxygen therapy, inhaled bronchodilators and systemic steroids, fulfilled one of the following criteria: pH lower than 7.35 with pCO_2_ higher than 47 mmHg; dyspnoea at rest with respiratory rate higher than 23 breaths/min; use of accessory respiratory muscles or paradoxical abdominal breathing [7]. Exclusion criteria were: refusal of NIV, deep hypercapnic coma; facial deformity; upper gastrointestinal bleeding; tracheal stenosis; acute ischemic heart disease; psychomotor agitation requiring sedation or need for urgent intubation due to cardiac or respiratory arrest [7].

Patients were ventilated with Trilogy A100 (Phillips Respironics, Murrysville, Pennsylvania, USA) using full face or oro-nasal masks. The following ventilator settings were initially used: BPAP S/T AVAPS mode with IPAP min/max 16/30cmH_2_O, EPAP 6-8cmH_2_O, respiratory back-up rate of 16/min, target volume of 5-7 ml/kg. Adjustments of these parameters were made by the treating physician based on blood gas measurements, oximetry, patient tolerance, and patient-ventilator synchrony, according to our internal NIV protocol. NIV was initially administered as long as necessary to maintain a stable pH of ≥7.35, followed by a gradual decrease of NIV usage time over the following days. NIV weaning success was defined as the lack of need for NIV for more than 48 h. NIV failure was defined as either endotracheal intubation or in-hospital death.

mIPAP and hours/duration of NIV were automatically recorded by the ventilator and digitally analyzed using Direct View software (Phillips Respironics, Murrysville, Pennsylvania, USA). We have derived from these recordings the number of hours of NIV in the first 24, 48 and 72 h (i.e. dNIV-24 h, dNIV-48 h and dNIV-72 h), the number of days with NIV (i.e. days of NIV) and the mean inspiratory pressure attained by the ventilator in VAPS-BPAP mode during the first 72 h (i.e. mIPAP-72 h).

### Patient variables

The following variables were considered to be possible predictors of NIV settings, duration, and outcome: age, BMI, lung function (FEV_1_ and FEV_1_/FVC ratio), arterial pH and pCO_2_, creatinine clearance (CC), and echocardiographic data and were consequently recorded. We also recorded the proposed outcomes: length of stay, NIV failure, and in-hospital mortality.

Spirometry was undertaken before discharge according to ATS/ERS guidelines [8] with a microQuark spirometer (Cosmed Srl, Rome, Italy). In patients that died without spirometry performed during the respective hospital admission, we have taken the last recorded spirometry. Arterial blood gases (pH, pCO_2_) were measured before (t0) and at 1–2 h (t1) and 4–6 h (t2) after the start of non-invasive ventilation with a blood gas analyzer.

### Comorbidities

Left heart failure [9] and kidney disease [10] are associated with increased risk of weaning failure in mechanically ventilated patients. Consequently, they might influence the implementation and the outcome of NIV in AECOPD, prompting us to include them among the studied variables.

Creatinine clearance (CC) was calculated using the MDRD study equation [11] based on admission serum creatinine. Kidney disease was defined as CC < 60 ml/min, either acute or chronic.

Echocardiography was performed within 72 h after admission. Left heart dysfunction (LHD) was defined by the presence of at least one of the following: reduced left ventricle ejection fraction (< 40%), left ventricular hypertrophy (interventricular septum and/or posterior wall thickness > 10 mm), left atrium diameter > 39 mm in women and > 41 mm in men, moderate or severe mitral regurgitation, increased left ventricular filling pressure (restrictive or pseudo-normal trans-mitral flow pattern).

### Statistical analysis

The analysis was performed using SPSS software 19.0 (SPSS Inc., Chicago, IL, USA). The Pearson product-moment correlation and the point-biserial correlation were used in univariable analysis. The result was reported as the correlation coefficient (r). Variables with a *p*-value lower than 0.01 on univariable analysis were considered eligible for multivariable analysis. To avoid multicollinearity, we examined correlations between all eligible variables using Pearson’s product-moment correlation. Variables that were likely to be similar and dependent on the others (i.e., a strong association between independent variables) were excluded. Eligible variables were entered into forward stepwise regression models (standard multiple or binomial logistic regression) [12]. Variables with a p-value lower than 0.05 were considered statistically significant, and their predictive value was reported as the regression coefficient (b) with a 95% confidence interval (CI).

## Results

**Fig. 1:**
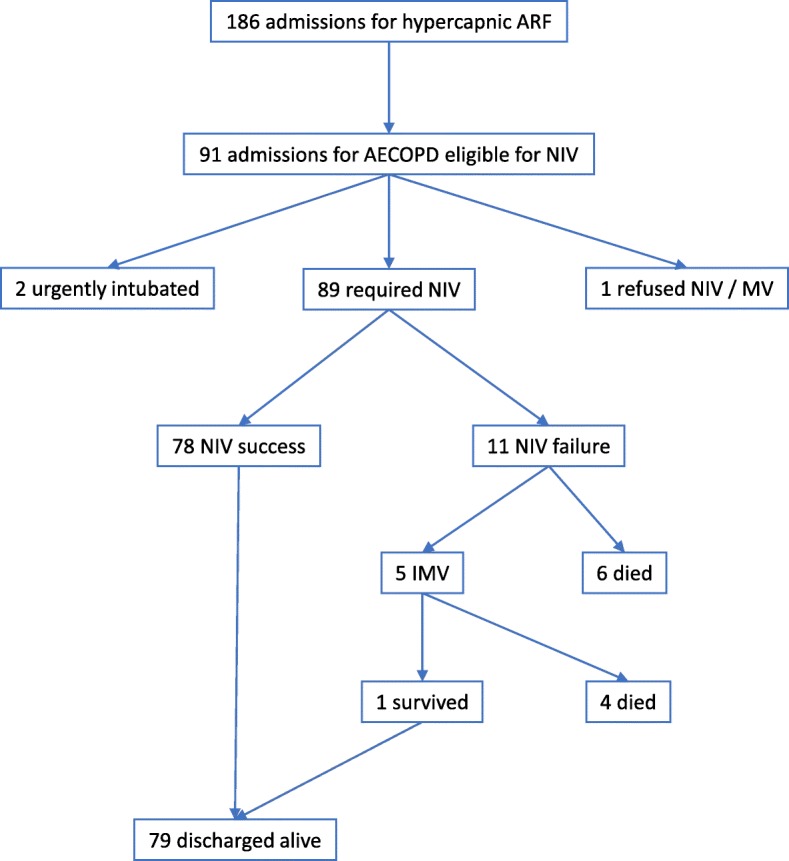
Patient flow chart. Legend: AECOPD acute exacerbation of COPD; NIV noninvasive ventilation; IMV invasive mechanical ventilation; ARF acute respiratory failure

A total of 89 patients (45 males, mean age 67.6 years) were included in the study; 2 patients were excluded due to emergency intubation at admission, and one refused any ventilatory support (Fig. 1).

Patient characteristics are presented in Table 1. Patient outcomes are presented in Table 2.

**Table 1:** Patient characteristics

	Mean (SD)	Range
Age (years)	67.6 (10.1)	46–97
BMI	28.9 (7.0)	17–43
FEV1% predicted	32.7 (10.8)	13–64
FEV_1_/FVC	48.0 (11.3)	19–66
pH-t0	7.26 (0.06)	7.03–7.41
pCO_2_-t0 (mmHg)	80.1 (19.7)	46.2–151

Legend:

BMI body mass index, FEV1 forced expiratory volume over 1 s, FVC forced vital capacity, pCO2 arterial partial pressure of carbon dioxide

t0 = before the start of NIV

**Table 2:** Patient outcomes

mIPAP-72 h (cmH_2_O)	17.5 *±* 4.4
pH-t1	7.30 ± 0.06
pH-t2	7.33 ± 0.07
pCO_2_-t1 (mmHg)	75.3 ± 16.8
pCO_2_-t2 (mmHg)	71.9 ± 14.7
dNIV-24 h (hours)	13.8 *±* 5.5
dNIV-48 h (hours)	23.2 *±* 10.3
dNIV-72 h (hours)	30.3 *±* 14.5
Days of NIV (days)	5.1 *±* 3.4
Length of stay (days)	8.6 *±* 6.2
NIV failure	11 (12.4%)
Mortality	10 (11.2%)

Legend:

NIV noninvasive ventilation, mIPAP-72 h mean inspiratory pressure in the first 72 h

t1 / t2 = 1-2 h and 4-6 h after the start of NIV

dNIV-24 h / -48 h / -72 h = duration of NIV in hours in the first 24 h, 48 h and respectively 72 h

data presented as mean n ± SD or n (%)

Thirteen patients (14%) met the criteria for kidney disease. Echocardiography was performed in 39 patients; 24 patients (61.5% of the subgroup with echocardiography) had LHD according to the established criteria.

NIV failure was recorded in 11 patients (12,4%). All patients with NIV failure were intubated; however, 6 of these patients had respiratory arrest during NIV, were intubated and died during resuscitation. The remaining five patients were intubated and transferred to ICU; only one patient survived and was successfully discharged at home. Thus 10 patients with NIV failure died (11.2% in-hospital mortality).

### Main outcome

NIV failure was correlated with days of NIV (*r* = 0.372, *p* < 0.001), LOS (*r* = 0.432, *p* < 0.001), in-hospital mortality (*r* = 0.947, *p* < 0.001), kidney disease (*r* = 0.224, *p* < 0.05) but not with age, FEV_1_, pH, pCO_2_ or LHD. Because of the low number of patients with NIV failure, further multivariable analysis was not appropriate. Neither dNIV-72 h nor mIPAP-72 h had any influence on NIV failure.

### Secondary outcomes

LOS was found to be positively correlated with dNIV-72 h, dNIV-48 h, days of NIV (*p* < 0.01) and with age (*p* < 0.05) but not with other variables. To avoid multicollinearity, we chose dNIV-72 h to be further entered in the regression model. In multivariable analysis, both dNIV-72 h and days of NIV were independent predictors for the length of hospital stay (Table 3). Thus, each increase of 7 h in NIV duration in the first 72 h resulted in approximately one more day of hospital stay.

**Table 3:** Variables associated with Length of Stay

Variable	Univariable analysis		Multivariable analysis		
	r	p	b	95%CI	p
dNIV-72 h	0.510	*p* < 0.001	0.13	0.04–0.21	0.003
dNIV-48 h	0.381	*p* < 0.001			
Days of NIV	0.542	*p* < 0.001	0.68	0.31–1.05	< 0.001

Legend:

dNIV-48 h / -72 h = duration of NIV in hours in the first 24 h, 48 h and respectively 72 h

Days of NIV = number of days with non-invasive ventilation

r correlation coefficient; b regression coefficient; 95%CI 95% confidence interval

In-hospital mortality was positively correlated with LOS (*r* = 0.437, *p* < 0.001), days of NIV (*r* = 0.357, *p* < 0.01) and FEV_1_ (*r* = − 0.220, *p* < 0.05). A strong association was identified in single variable regression analysis between days of NIV and mortality with an odds ratio of 1.27 (95% CI 1.07–1.5, *p* = 0.005); consequently, each additional day with NIV would result in a 27% increase in mortality rate.

BMI, pH and pCO_2_ levels, mIPAP-72 h, and presence of LHD or kidney disease had no influence on mortality or length of hospital stay in our cohort.

Mean hours of NIV within the first 24, 48 and 72 h were: 13.8 (SD = 5.5), 23.2 (SD = 10.3) and 30.3 (SD = 14.5) respectively.

We found dNIV-72 h to be associated with several variables in univariable analysis (see Table 4). pH-t0, pH-t1, pH-t2, and presence of LHD were eligible to be entered in the regression model (i.e., with a *p* < 0.01). Because of significant and strong correlations between the three pH variables, only pH-t2 (*r* = − 0.364 – strongest correlation to dNIV-72 h) together with the presence of LHD were chosen to be entered in the standard multiple regression and both variables were confirmed to be independent predictors of dNIV-72 h with a regression coefficient of 11.5 (95% CI: 3.5–19.5) for LHD and − 73.1 (95% CI: − 127.4 - -18.8) for pH-t2.

**Table 4:** Association of different variables with dNIV 72 h or mIPAP-72 h in univariable analysis

	dNIV-72 h		mIPAP-72 h	
Variable	r	p	r	p
Age	0.231	0.03	0.218	0.04
BMI	ns	ns	**0.501**	**< 0.001**
FEV1% predicted	ns	ns	0.227	0.03
FEV1/FVC	ns	ns	**0.319**	**0.002**
LHD^a^	**0.412**	**0.009**	ns	ns
Kidney disease	0.230	0.04	ns	ns
pH-t0	−0.323	0.002	ns	ns
pH-t1	−0.337	0.001	ns	ns
pH-t2	**−0.364**	**< 0.001**	ns	ns
pCO2-t0	ns	ns	**0.357**	**0.001**
pCO2-t1	ns	ns	0.332	0.001
pCO2-t2	ns	ns	0.264	0.013

Legend:

^a^subgroup of 39 patients

variables in bold have been introduced in multiple regression analysis, see text

BMI body mass index; LHD left heart dysfunction; FEV1 forced expiratory volume over 1 s, FVC forced vital capacity; mIPAP-72 h mean inspiratory pressure in the first 72 h; pCO2 arterial partial pressure of carbon dioxide; ns not significant

t0 / t1 / t2 = before, and 1-2 h, respectively 4-6 h after the start of NIV

Thus, the presence of LHD and a pH lower with one decimal at 4–6 h significantly and independently prolonged NIV duration in the first 72 h by approximately 11 and 7 h respectively. BMI, FEV_1,_ and pCO_2_ values did not influence NIV duration during the first 72 h.

Mean IPAP in the first 72 h (mIPAP-72 h) was 17.5 (SD = 4.4). In univariable analysis, mIPAP-72 h was found to be associated with several variables (see Table 4). BMI, FEV_1_/FVC, pCO2-t0, and pCO2-t1 were eligible to be entered in the regression model (i.e., *p* < 0.01). With a strong correlation between pCO2-t0 and pCO2-t1, only pCO-t0 (strongest association to mIPAP, *r* = 0.357) together with BMI and FEV1/FVC were entered in standard multiple regression. BMI and pCO2-t0 were confirmed to be independent predictors of mIPAP-72 h with a regression coefficient of 0.26 (95% CI: 0.14–0.39) and 0.06 (95% CI: 0.02–0.10), respectively. Thus, each increase in BMI by 4 kg/m^2^ and in pCO_2_ by 16 mmHg resulted in an approximate increase in mIPAP-72 h by one cmH_2_O.

pH values and the presence of either kidney disease or LHD did not influence the inspiratory pressures needed by these patients in the first 72 h of NIV.

We did not find any variables to be associated with both dNIV-72 h or mIPAP-72 h at *p* < 0.01, suggesting that these two characteristics have independent determination.

## Discussion

We found a 12% rate of NIV failure and 11% mortality rate in our population of patients with AECOPD treated with non-invasive ventilation. These figures are at the lower end of the ones reported in other studies where NIV failure rate was between 9 and 50% [6].

Our data shows that LOS is associated with in-hospital mortality. This finding is backed up by previously published data that found LOS to be a predictive variable for in-hospital and up to 6 months mortality after admission to ICU in AECOPD patients [13]. Moreover, we observed an association between in-hospital mortality and days of NIV. While we did not find any prior studies regarding this relation in AECPOD patients, a strong association is described in non-COPD patients with ARF [14]. Likewise, patients with AECOPD requiring more days of invasive mechanical ventilation have an increased risk of poor outcome [15].

Determinants of outcomes of AECOPD patients treated with NIV that we investigated and are common to other studies are initial arterial blood gases, age, BMI, FEV1, and comorbidities.

The pH level has been reported to be a critical prognostic factor [16]. Confalonieri et al. examined predictive factors related to NIV failure in a large multicentric study which included 1033 patients with severe AECOPD. In their study, pH values, respiratory rate, GCS, and APACHE II severity score recorded at t0 and t1 were found to be independent predictive variables for NIV failure. These variables were then used to build a prognostic model for NIV failure, which proved to have high accuracy, especially at t1 when assessed on an independent sample of patients. Specifically, a pH < 7.25 at t1 was an important predictive indicator of NIV failure [17]. Despite including patients with severe acidosis (pH < 7.15), we did not identify any relation between pH and NIV failure or mortality. On the other hand, this would support a recent recommendation that severe acidosis should not preclude an NIV trial in AECOPD [7].

Although pCO2 has been reported to be associated NIV failure in non-COPD ARF [18], we did not find any such association in AECOPD patients but instead observed that pCO_2_ – t1 is an independent predictor for mIPAP-72 h which has not been reported before.

Older age is reported to be a predictor for in-hospital or long-term mortality in AECOPD patients [19]. In our study older patients required more days of NIV and a longer LOS, had a more severe functional impairment and required higher inspiratory pressures but had the same outcome (i.e., NIV failure and in-hospital mortality) as younger patients. This result confirms our previous findings [20] and supports the current recommendation for NIV support regardless of age in AECOPD [7].

A low BMI < 20 is associated with higher mortality rates in AEOCPD patients admitted in the ICU for mechanical ventilation (invasive or NIV) [21]. In our study, BMI influenced NIV settings but not NIV failure or mortality, suggesting that NIV might be similarly effective irrespective of BMI values.

In the univariable analysis, we found FEV_1_ to be negatively correlated with in-hospital mortality. We could not find any data in the literature regarding the impact on FEV_1_ on in-hospital mortality in AECOPD with hypercapnic ARF, but outside the hospital setting FEV1 is a well-known predictor of general mortality.

Heart failure (HF) is present in more than 20% of patients with COPD [22]. Despite this, few data exist regarding the impact of HF on the outcome of severe AECOPD. Given this context, we specifically looked for echocardiographic signs for LHD in a subgroup of our population. We observed that, despite increasing the amount of NIV within the first 72 h, the presence of LHD did not influence the length of hospital stay, NIV failure, or mortality rate. To our knowledge, there is only one other study that looks at the influence of left heart dysfunction in AECOPD, requiring NIV who reported similar findings [23].

Whether the presence of chronic kidney disease (CKD) is of any consequence in patients with severe AECOPD is still in debate [24]. Our results show that patients with acute or chronic kidney disease were older, were prone to NIV failure, required more hours of NIV very likely because of the associated metabolic acidosis with lower pH at t0, t1, t2. Despite all these correlations, the presence of kidney disease did not influence in-hospital mortality.

While there is no previous data on this subject, we found that the amount of NIV applied within the first 72 h and the amount of IPAP used in the first 72 h have no impact on NIV failure nor on mortality. Instead, NIV-72 h does influence the number of days of NIV and LOS. This data can be considered one of the original findings of this study.

Moving on to the secondary outcomes, although the number of studies regarding NIV in AECOPD is large there is no other study to our knowledge that specifically looks at factors that influence NIV duration and settings in the first 72 h of hospital admission.

Regarding NIV settings, one common reason for NIV failure is insufficient pressure support, and inadequate IPAP is often used in AECOPD [25, 26], and there is no published data regarding factors that directly influence IPAP. Thus, we decided to look for such factors, and we found that BMI and baseline pCO2 need to be considered when initially choosing NIV settings.

Concerning NIV duration, current guidelines recommend maximization of NIV duration during the first 24 h, followed by tapering NIV according to pH, pCO_2_ levels and tolerance in the next 2 to 3 days [7]. After the first 24 h, the optimal amount of NIV and the right time to withdraw it has not been examined in published trials. Thus, a study of NIV duration within the first 72 h is justified.

In our study, dNIV-72 h was determined by the pH level and the presence of left heart dysfunction, while not being influenced by other common variables such as BMI, FEV1, or pCO2 levels. Both the pH level and the presence of LHD significantly and independently increased the need for NIV in the first 72 h.

It is interesting to note that dNIV-72 h and mIPAP-72 h had different predictors, thus being independent of each other. Consequently, different characteristics will be considered when setting inspiratory pressure than when setting the duration of NIV.

We consider that this study has limitations. First, in comparison to many of the studies mentioned above, we had a low number of enrolled patients which may limit its external validity. Second, given our inability to record accurate pO2/FiO2 at admission, we were unable to calculate any severity score (i.e., SAPS or APACHE) and thus its implications on outcomes as other studies have reported.

## Conclusion

In our cohort of AECOPD patients, NIV failure and in-hospital mortality were not influenced by age, BMI, the presence of left heart dysfunction and the arterial blood gases values in the first 6 h of NIV. These findings support the extensive use of NIV in AECOPD irrespective of these characteristics, similar to current recommendations.

The total amount of NIV hours needed in the first 72 h was higher in patients with more severe acidosis during the first 6 h of NIV, and in patients with left heart dysfunction.

Mean IPAP needed for NIV in AECOPD patients is proportional to the BMI and to pCO2 (which can be used to predict mIPAP) and is not related to the level of lung function or the severity of acidosis.

## Data Availability

The datasets used and analyzed during the current study are available from the corresponding author on reasonable request.

## Abbreviations

95%CI: 95% confidence interval
AECOPD: Acute exacerbation of chronic obstructive pulmonary disease
ARF: Acute respiratory failure
b: Regression coefficient
BMI: Body mass index
BPAP: Bilevel positive airway pressure
CC: Creatinine clearance
CKD: Chronic kidney disease
COPD: Chronic obstructive pulmonary disease
EPAP: Expiratory positive airway pressure
ETI: Endotracheal intubation
FEV1: Forced expiratory volume over 1 s
FiO2: Fraction of inspired oxygen
FVC: Forced vital capacity
HF: Heart failure
ICU: Intensive care unit
IPAP: Inspiratory positive airway pressure
LHD: left heart dysfunction
LOS: length of hospital stay
LVH: left ventricular hypertrophy
min: Minute
mIPAP: Mean inspiratory pressure
mIPAP-72 h: Mean inspiratory pressure in the first 72 h
NIV: Noninvasive ventilation
dNIV-24 h/− 48 h/− 72 h: Duration of noninvasive ventilation in hours in the first 24 h, 48 h and respectively 72 h.
pCO2: Arterial partial pressure of carbon dioxide
pCO2-t0/−t1/−t2: Arterial partial pressure of carbon dioxide at admission, at 1–2 h and respectively 4–6 h after start of noninvasive ventilation
pH-t0/−t1/−t2: ph level at admission, at 1–2 h and respectively 4–6 h after start of noninvasive ventilation
pO2: Arterial partial pressure of oxygen
r: Correlation coefficient
S/T: Spontaneous/timed ventilation mode
SD: Standard deviation
VAPS: Volume assured pressure support ventilation
